# Supplementary material for: HCN2 Channels in the Ventral Hippocampal CA1 Regulate Nociceptive Hypersensitivity in Mice
Source: Int J Mol Sci. 2023 Sep 7;24(18):13823. doi: 10.3390/ijms241813823 (PMC10531460; doi:10.3390/ijms241813823)
Supplement: Supplementary file 1 [file ijms-24-13823-s001.zip › ijms-2528095-supplementary.pdf]

Supplementary Material

1.1 Figures S1

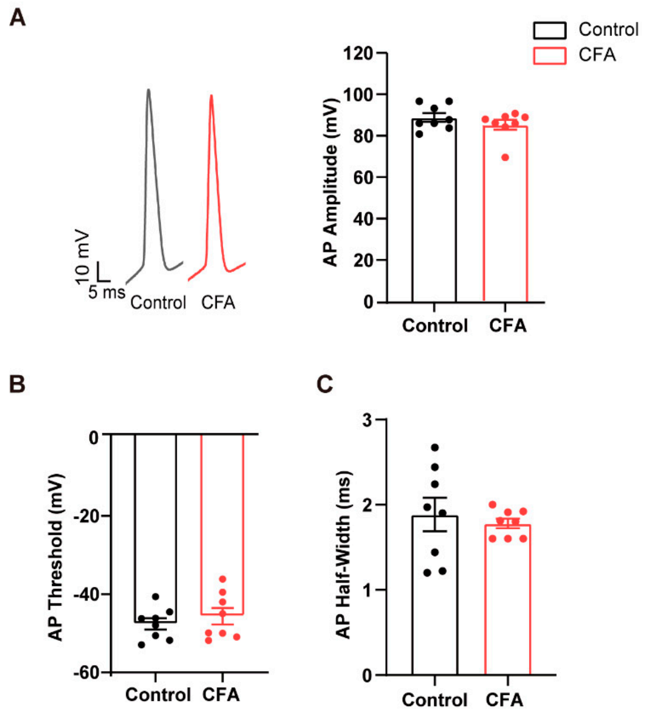

Figure S1. Associated data for Figure 1. The amplitude (A), threshold (B) and half-width (C) did not change in vCA1 pyramidal neurons of mice with CFA-injection, unpaired two-tailed Student's t-test.

1.2 Figures S2

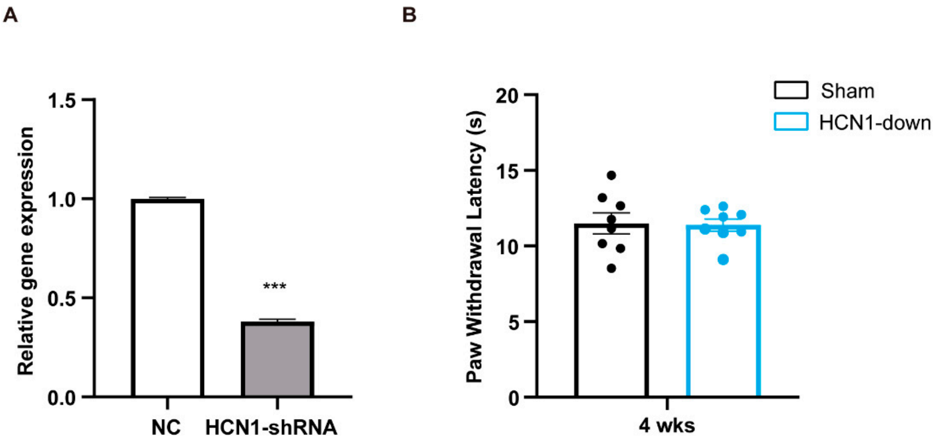

Figure S2. HCN1 knock-down in the vCA1 did not affect physical thermal pain thresholds. A. Relative levels of HCN1 determined by real-time RT-PCR in vitro, expressed as a fold change relative to NC (negative control). n = 3 in each group. *** $p < 0.001$ vs NC, unpaired two-tailed Student's t-test. B. Knock-down of HCN1 in vCA1 did not affect the physical thermal pain thresholds in mice. two-way ANOVA with Sidak's multiple comparisons tests.

1.3 Figures S3

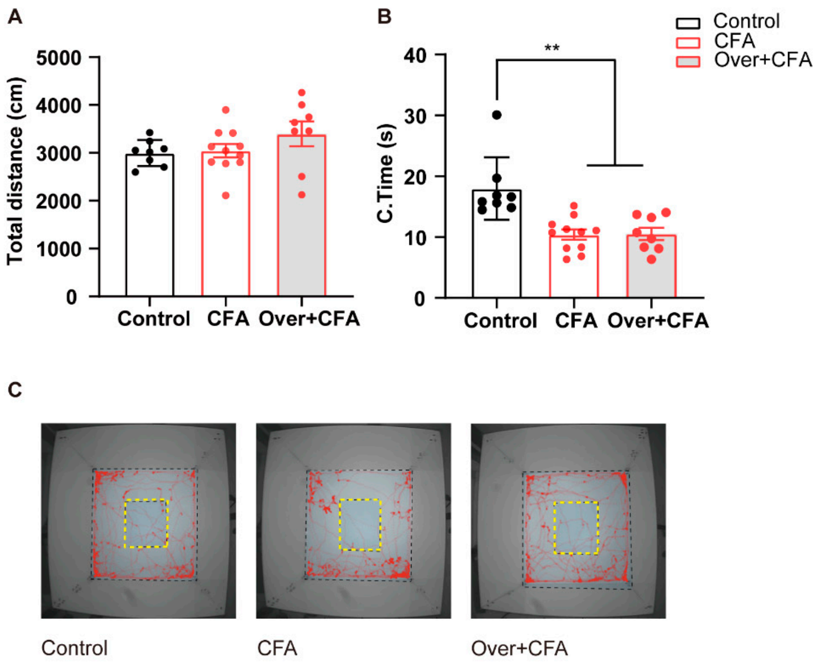

Figure S3. Up-regulation of HCN2 in vCA1 shows limited effects on locomotion in CFA-injected mice. (A) Up-regulation of HCN2 in vCA1 had limited effects on total distance traveled in the field in CFA-injected mice. *n* = 8 in each group. One-way ANOVA with Sidak's multiple comparisons tests. (B) Up-regulation of HCN2 in vCA1 had limited effect on time spent in the center area of the field in CFA-injected mice. (C) Representative exploratory tracks (red polylines) in the open field. Yellow dash lines indicate open arms.
